# Effects of Traumatic Brain Injury on the Orexin/Hypocretin System

**DOI:** 10.1089/neur.2024.0111

**Published:** 2025-04-21

**Authors:** Rebecca T. Somach, Miranda M. Lim, Akiva S. Cohen

**Affiliations:** ^1^ CHOP At Penn Medicine Hospital of The University Pennsylvania, USA.; ^2^ Oregon Health and Science University, Portland, Oregon, USA.; ^3^ VA Portland Health Care System, Portland, Oregon, USA.; ^4^ Children’s Hospital of Philadelphia, Philadelphia, Pennsylvania, USA.; ^5^ University of Pennsylvania, Philadelphia, Pennsylvania, USA.

**Keywords:** hypocretin, orexin, sleep disruption, traumatic brain injury

## Abstract

Traumatic Brain Injuries (TBIs) are known to cause a myriad of symptoms in patients. One common symptom after injury is sleep disruptions. One neuropeptide system has been studied repeatedly as a potential cause of sleep disruptions after TBI- the orexin/hypocretin system. Orexin promotes wakefulness and arousal while disrupting the orexin system causes increased sleepiness and narcolepsy. Studies of TBI in human and animal subjects have shown that TBI affects the orexin system. This review serves as an overview of how TBI affects the orexin/hypocretin system, including structural and functional changes to the neurons after injury. This review is the first to include studies that examine how TBI affects orexin/hypocretin receptors. This review also examines how sex is accounted for in the studies of the orexin system after TBI.

## Introduction

A traumatic brain injury (TBI) is defined as a bump or blow to the head or a penetrating injury.^1^ TBIs also include injuries from rotational forces and blast injuries that are caused by the pressure wave of nearby explosives moving the brain inside the skull. TBIs are common, with an estimated 1.7 million TBIs occurring yearly in the United States alone,^2^ and an estimated 27–69 million TBIs taking place worldwide each year.^3^ The trauma from TBI results in two phases of injury, primary and secondary injury.^4^ Primary injuries are the direct result of the mechanical forces of the injury. This includes the deformation of tissue, shearing of axons, and cell death caused by the forces generated by the TBI. Secondary injuries are the ongoing cellular processes that occur as a result of mechanical trauma,^5^ such as inflammation,^6^ and changes in cellular metabolism as well as other cellular cascades that can lead to cell death and degeneration.

TBIs lead to a variety of symptoms that disrupt the body and brain physically, cognitively, and emotionally.^7–9^ Sleep disruption is a symptom of TBI that can affect other known outcomes of injury because a lack of proper sleep disrupts healthy living.^10–15^ TBI patients experience sleep disorders at higher rates than in the general population,^16^ suggesting that sleep disorders can occur as a result of head injury. When examining sleep disorders after TBI, much TBI research has been devoted to studying orexin/hypocretin, a neuropeptide involved in the regulation of wakefulness. Both hypocretin and orexin are appropriate names for the same neuropeptide, but the term orexin will be used in this review.

In sleep-wake physiology, orexin maintains wakefulness and arousal, as well as regulates sleep stability.^17–21^ In 2017, we wrote a comprehensive review of many aspects of sleep and TBI across rodent and human species and we included one section on TBI and orexin and noted its importance in the sleep and TBI space.^22^ The purpose of the current review is to provide a focused, 7-year update on the state of research on TBI and orexin. This review will expand upon studies that report how TBI affects the orexin system directly, including effects on orexin neuropeptide, orexin neurons, and orexin neuron physiology and function. There is some literature that describes treatment options that promote or inhibit orexin peptide or activity in the brain after TBI.^23–25^ While treatments are important for the future of orexin research in brain injury, they are outside of the scope of this review. Instead, we will turn our attention to the neuropeptide orexin itself and how TBI may lead to deleterious consequences in the orexin system and thereby affect sleep-wake function. In addition to the function of the orexin neurons themselves, this review will discuss changes to orexin receptors after injury, which could be an indication of the way the orexin system responds to injury as a whole. Finally, this is the first review to address TBI, the orexin system, and sex differences which are often understudied in TBI literature.

Orexin neuropeptide is produced in the brain by orexin neurons located in the lateral hypothalamus and was discovered by two separate groups in 1998.^26,27^ Even though orexin neurons are naturally low in number and most orexin neuron cell bodies are concentrated in the lateral and posterior hypothalamus, they exert an outsized influence on animal behavior. Orexin neurons are involved in the behaviors of wakefulness, feeding, attention, reward, homeostasis, cognition, and stress.^20,28–30^ A trait that many of these behaviors share is their association with motivational arousal,^31^ that is, orexin neurons respond to salient environmental stimuli and allow an animal to respond appropriately.

Often TBI research focuses on orexin neurons because of their ability to regulate sleep and wakefulness. Orexin neurons are both necessary and sufficient for wakefulness. If orexin is injected into the brain, it causes an increase in wakefulness.^32,33^ Without orexin or with altered orexin receptors, humans and animals experience narcolepsy,^34–39^ which is a state of spontaneously falling asleep at inappropriate times. Orexin knockout animals demonstrate the same amount of sleep overall compared to normal animals but have shorter bouts of sleep with more transitions between behavioral states.^39–41^ These experiments provide evidence that orexin is at the center of regulating the switch between wakefulness and sleep and recent direct evidence confirms that orexin neuropeptide inhibits sleep-promoting neurons.^21^ While orexin helps regulate sleep and wake, other neuropeptides also contribute to sleep and wake regulation.^42,43^ Studies of TBI and other regulatory neuropeptides include histaminergic neurons which promote wakefulness and can be reduced after TBI,^44–46^ cholinergic neurons that are reduced after TBI,^45^ and melanin-concentrating hormone (MCH) neurons that promote sleep and are intermingled with orexin neurons in the lateral hypothalamus and are reduced after injury.^46,47^ Since orexin neurons are not the only regulatory neuropeptides in the arousal system, it is important to determine if damage to the orexin system has a role in sleep disruption observed after TBI.

This question was addressed by a group of researchers who studied sleep behavior after TBI in mice with a knockout for orexin neuropeptide.^48^ Both the knockouts and control animals were given a moderate TBI and compared with sham-injured animals. The injury decreased wakefulness in the control animals. The orexin knockout itself had shortened bouts of wakefulness, but the TBI did not change the distribution of wake further. The authors of this study concluded that the animals needed to have orexin present in order to observe TBI-induced sleep deficits. This evidence suggests that orexin contributes to the difference between the sleep of sham-injured and injured animals after injury.

Another reason to focus on orexin neurons after injury is that they may have a sensitivity to the effects of TBI. A good comparison is to examine studies of orexin and MCH after TBI. The two neuronal subtypes are both located in the same space in the lateral hypothalamus and individual neurons are intermingled with one another, suggesting that in one brain the neurons would be exposed to similar forces during a TBI. Damage due to the primary injury of TBI, which is the physical component of injury that includes stretch, shearing, and rotational forces, should be shared similarly by both types of neurons due to them being physically next to one another in the lateral hypothalamus. MCH neurons show no significant changes after injury in some studies.^44,45,49^ In one of these studies,^45^ the number of orexin neurons decreased without significant changes in the MCH neurons, though all animals shared the same injury parameters and were studied by the same group of researchers. This suggests that at the same injury severity, orexin neurons may be more vulnerable to the effects of the TBI as compared to MCH neurons. Alongside the evidence from the knockout studies, this suggests that orexin neurons are a class of neurons that should be focused on after TBI. While orexin neurons are notable for a potential sensitivity after injury, it is the case that some studies have evidence of reduced numbers of MCH neurons after injury. However, this outcome was observed after severe TBI,^46,47^ and a severe enough injury may damage all neurons regardless of sensitivity to injury. While orexin neurons have been frequently studied after TBI, this possibility warrants further study of how the injury affects other neuronal subtypes involved in the sleep and wake systems.

## Studying Orexin Following TBI—Human Studies

The first studies that measured orexin in cerebral spinal fluid (CSF) from humans occurred in 2000 and 2001, shortly after the discovery of orexin neuropeptide.^37,50,51^ Ripley et al. 2001, measured orexin in patients with narcolepsy and a wide range of neurological conditions.^51^ One of these conditions was craniocerebral trauma, now referred to as TBI. It would follow that TBI is a known risk factor for narcolepsy, known as post-traumatic narcolepsy.^52–54^ Six out of seven patients with craniocerebral trauma in the Ripley et al. 2001 study demonstrated low levels of orexin.^51^ In four of these patients, cerebral spinal fluid was collected 5 to 11 days after the trauma. One of the patients had CSF collected 7 months after injury but still had low levels of orexin. In this study, some patients with neurological conditions, including patients with intracranial tumors, Guillain-Barre syndrome, central nervous system infections, and acute lymphocytic leukemia, had a statistically significant drop in orexin levels. However, patients with neurodegenerative diseases such as Parkinson’s disease, Alzheimer’s disease, and multiple sclerosis had normal orexin levels, suggesting that not all neurological conditions damage orexin neurons or reduce their ability to produce orexin peptides. Another study in children observed a similar outcome.^55^ Of the children studied, 3/3 subjects with head trauma had low or intermediate levels of orexin. The sample size in this study is small, but it is notable that all three subjects had a decrease in orexin levels when this was not the case for other disorders measured.

In 2005, a study was conducted to measure orexin after TBI in the CSF of 44 TBI patients.^56^ In this group of patients, 37/44, or 84%, had low orexin levels. Of the patients, 30/31 severe TBI patients and 7/8 patients with moderate TBI had low levels of orexin. This was measured acutely at 1 to 4 days after injury. The same researchers then examined orexin levels in TBI patients 6 months after injury.^57^ In this study, orexin levels were measured in 27 patients when they were first admitted 1–4 days after injury and 21 patients had follow-up measurements 6 months later. Most patients had low orexin levels in the acute phase of injury; 13/27 had undetectable levels of orexin, 12 had low levels, and 2 had normal levels where 320 pg/mL was considered the cutoff for low levels of CSF orexin. At the 6-month mark, most patients had increased orexin compared to the original time point, with 17/21 patients observed to have normal levels of orexin and 4 patients that had low levels of orexin. Patients with excessive daytime sleepiness (EDS) at 6 months after injury had lower orexin levels compared to patients who did not display EDS. This demonstrates a link between the sleepiness of patients and low levels of orexin in their CSF. Furthermore, this indicates that it is possible for certain patients to experience sleep deficiencies at times far past the initial injury, just as the patient did 7 months after injury as reported in the study done by Ripley et al. 2001.^51^ These human studies provide evidence that TBI can affect orexin production.

In order to address reasons for low CSF orexin levels, several studies have quantified orexin neurons in humans after TBI.^46,47^ Baumann et al. 2009 found that in 4 TBI patients, the number of hypocretin neurons was decreased by 27% compared to controls.^47^ In Valko et al. 2015, 12 TBI patients experienced a 21% loss of orexin neurons compared to controls.^46^ One additional study, Kousi et al. 2021 also found reduced orexin neurons in humans after TBI,^58^ but this study lacked adequate controls for us to further evaluate in our review. A caveat of any studies that measure the number of orexin neurons in humans is that they are measured after severe TBI. These studies are unable to determine what happens to orexin neurons immediately following a mild or moderate TBI. Patients with mild TBI still experience sleep disorders,^59,60^ and show decreased orexin in the CSF,^56,57^ but need to be explored through other means aside from human-based research.

## Studying Orexin Following TBI—Animal Studies

Animal research has been used to model many of the physiological and behavioral consequences of TBI in humans,^61–63^ and allow for studies that would not be possible in humans. Animal models recapitulate sleep phenotypes of disordered sleep in humans, especially that of EDS.^22,44,45,48,49,64–73^ Some studies report a disordered sleep phenotype of increased wakefulness, more latency to sleep, or insomnia in animals,^65,70–72^ which are also phenotypes of sleep disorders in human TBI patients.

**FIG. 1.**
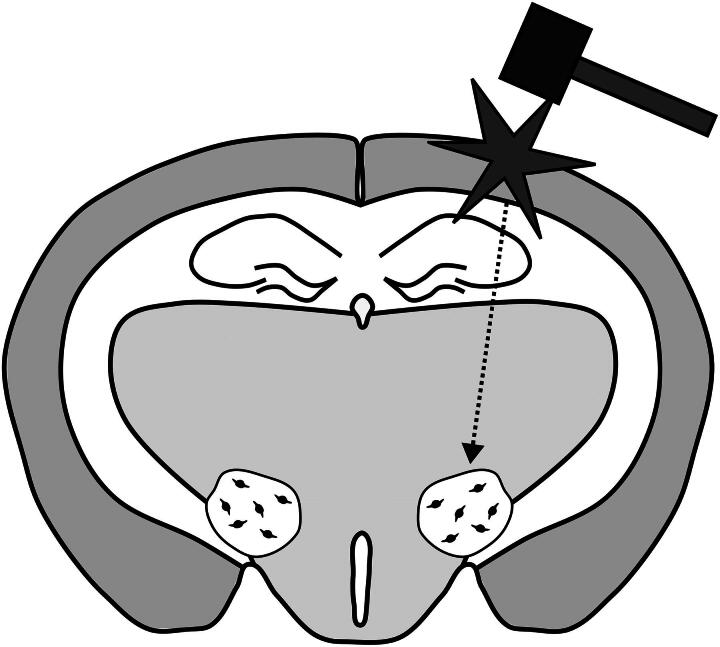
Schematic of typical location of a model of lateral traumatic brain injury and the location of lateral hypothalamus and orexin neurons in a rodent brain. The figure depicts a rodent brain. The mallet and star represent a traumatic brain injury. The injury depicted is the location targeting in models of lateral injury, but some rodent models will induce the injury at the midline. The dotted arrow represents the distribution of force that may occur through the brain following a TBI. The small black circles are orexin neurons that are distributed randomly within the lateral hypothalamus. The figure is for illustrative purposes and is not to scale. TBI, traumatic brain injury.

In humans, TBIs are heterogeneous. Animal studies are designed to be replicable and homogeneous across animal subjects within a study. Several animal models of TBI have emerged.^63^ The models that have been used in animal studies of orexin include weight drop, controlled cortical impact (CCI), fluid percussion injury (FPI), and blast injury (See Table 1). In weight drop models, a weight is guided to hit the skull of the animal. This model can be done with or without a craniotomy to simulate open or closed head injury. FPI is accomplished by first creating a craniotomy and then releasing a piston from a pre-determined height to strike a fluid-filled cylinder creating a pressure wave leading to a pulse of pressure that impacts the intact dura matter. This method produces both focal and diffuse pressure and avoids skull fracture after injury. CCI is another open skull method that is conducted by performing a craniotomy and uses an impact device to directly hit brain tissue. This model reproduces tissue loss and deformation and produces a focal injury. In these three methods, the force is applied above or on the cortex (see Fig. 1). Blast injury simulates injuries that occur when the force of nearby explosives causes the brain to hit the inside of the skull. In blast injuries, a device, such as an advanced blast simulator, simulates an explosion using a pressure wave and produces a diffuse injury that does not specifically impact one part of the head. Each model produces a TBI with different qualities and it is important to be aware of this when comparing animal studies. Several studies have quantified the number of orexin neurons present after injury in animals, just as has been done in human subjects. Some studies report a significant reduction of orexin neurons after injury.^45,48,73^ Some studies show no significant differences in orexin neurons between injured and sham-injured animals.^44,67,69,71,74^ In our previous study, there was an increase in orexin neurons after injury.^49^ These differences could be due to many factors that are different between these animal studies of TBI. These studies differ in their methods of injury, the species of animal, the ages of the animals at the time of injury, the time between injury and measurement, and the severity of the injury. There is a summary of the details of these studies, and all those that measure orexin after TBI in Table 1. It is likely that these factors contribute to whether a study finds if orexin neurons are reduced after injury.

**Table 1.** Studies of Orexin Following TBI

Article	Finding related to orexin and TBI	Species	Age of subjects, or weight for animals if age not given	Sex of subjects	Method of injury	Severity of injury	The time between injury and measurement
Human studies							
Arii et al. 2004^55^	Orexin levels were intermediate in 1 patient (between 110–200 pg/mL) and low in 2 patients (less than 110 pg/mL)	Humans	15, 16, and 17 years old.	Men. 3 patients	Human subject	Moderate-severe	4 days, 1 month, and 10 months after injury.
Baumann et al. 2005^56^	Moderate-injured and severely injured patients had low levels of CSF hypocretin, defined as less than 320 pg/mL. 37 of 44 total patients had low levels of CSF hypocretin. 30 of 31 patients with severe TBI had low levels. 7/8 of moderate TBI patients had low levels of hypocretin. Mild injured patients had decreased hypocretin in CSF compared to spinal CSF controls.	Humans	Mean age of TBI patients: 36, range 17–69 years.	In TBI patients: both men and women. 32 men, 12 women	Human subject	Mild: 5 patients, moderate: 8 patients, severe: 31 patients.	1–4 days after injury.
Baumann et al. 2007^57^	25/27 patients had low levels of CSF hypocretin after acute injury (1–4 days post-injury). Low CSF hypocretin levels, as defined as less than 320 pg/mL, were found in 4 of 21 patients 6 months after TBI. Hypocretin levels 6 months after TBI were significantly lower in patients with post-traumatic excessive daytime sleepiness.	Humans	Mean age of TBI patients: 38, SD: 16 years, range 16–72.	For both sleep behavior and CSF studies: men and women. 96 patients, 75 men, 21 women. At the 6-month mark, 76 patients, 53 men and 12 women.	Human subject	Mild: 26 patients, moderate: 15 patients, severe: 24 patients	Acute TBI measurement within the first 4 days after TBI (whenever possible). Later results were taken 6 months later.
Baumann et al. 2009^55^	TBI subjects have fewer orexin neurons compared to controls, especially in the center of the orexin field.	Humans	Controls: 69 ± 18 years. TBI subjects: 51 ± 19 years.	All men except for one woman were in the control group. 4 TBI patients, 4 controls (3 men, 1 woman)	Human subject	Severe	7–42 days after severe TBI.
Ripley et al. 2001^51^	The injured population had lower levels of CSF hypocretin than controls. Low hypocretin is defined as less than 194 pg/mL. Controls: median 318 (range: 224–653). In injured subjects, the hypocretin median range was 147 (range: 129–294). 5/6 subjects had low levels of hypocretin.	Humans	N/A	N/A	Human subject	N/A	CSF was collected 5–11 days after trauma, and 7 months after trauma in 1 patient.
Valko et al. 2015^46^	Decreased number of orexin neurons in TBI subjects	Humans	Controls: 68 ± 12 years. TBI subjects: 70 ± 13.	Men and women. Controls 16 (9 men, 7 women). Injured 12 (9 men, 3 women)	Human subject	Severe	Mean survival after trauma of 27 ± 28 days (range: 7–85 days). The mean fixation times of TBI and control brains were similar. Fixation times varied from 1 month to 2 years.
Animal studies							
Dong and Feng 2018^79^	Increased orexin and orexin receptor expression after TBI.	Animals: Rats, Sprague-Dawley	Adult, 250–300 g	Males and females	Free-fall weight drop	Severe, comatose state. 400 g weight dropped from a height of 40–44 cm.	6 h, 12 h, 24 h
Dong et al. 2021^91^	Decreased expression of orexin receptors after injury.	Animals: Rats, Sprague-Dawley	Adult, 250–300 g	Males and females	Free-fall weight drop	Severe, comatose state. 400 g weight dropped from a height of 40–44 cm.	12 h
Du et al. 2022	Increased orexin and orexin receptor expression after injury.	Animals: Rats, Sprague-Dawley	Adult, aged 6–8 weeks, 250–300 g	N/A	Free-fall weight drop	Severe, 400 g weight dropped from a height of 40–44 cm.	24 h
Elliott et al. 2018^82^	Decreased axodendritic glutamate in the synapses onto orexin neurons in the hypothalamus.	Animals: Mice, C57/BL6J (Jackson)	5–7 weeks old, 20–25 g	Males	Lateral fluid percussion injury	Mild: 1.4 to 2.1 atm.	7 dpi
Huang et al. 2022^78^	TBI decreased the orexin content in brain tissue and increased the orexin receptor content compared to sham-injured controls.	Animals: Rats, Sprague-Dawley	7 weeks of age, 250–300 g	Males	Free-fall weight drop	400 g weight dropped freely from a height of 45 cm. Categorized severe from modified neurological severity scale.	3 dpi
Kang et al. 2024^90^	Orexin receptors are decreased after injury compared to sham-injured controls.	Animals: Rats, Sprague-Dawley	8 weeks of age at injury, 7 weeks, and 250–300 g when acquired	Males	Free-fall weight drop	40 g weight dropped from a height of 20 cm.	24 h
Lim et al. 2013^67^	The percentage of orexin neurons stained with cFos is reduced by TBI after a 3-h period of forced wakefulness. No changes in number of orexin neurons due to injury.	Animals: Mice, C57/BL6J (Jackson)	5–7 weeks old, 20–25 g	Males	Lateral fluid percussion injury	Mild: 1.4 to 2.1 atm.	4 weeks after TBI
Mihara et al. 2011^89^	Orexin 1 receptor immunoreactivity increased at the site of the injury in the cortex. Receptors first appeared at 6 h post-injury, then peaked at 1 day post-injury. Orexin receptor colocalized on microglia on day 1, then in microglia and neurons on day 7. Fibers with orexin immunoreactivity were seen in the penumbra of injury at the cortex on days 1 and 7 after injury.	Animals: Mice, C57BL/6 (SLC Japan)	8 weeks old	Males	Controlled cortical impact	Moderate: impact velocity of 5.82 m/sec, duration of 47 msec, depth of 1.2 mm, driving pressure of 73 psi.	0 h, 3 h, 6 h, 12 h, 1 dpi, 2 dpi, 4 dpi, 7 dpi
Noain et al. 2018^44^	No change in the number of hypocretin neurons after injury.	Animals: Rats, Sprague-Dawley	10–11 weeks old at injury. 300–350 g	Males	Closed skull TBI on the pre-frontocortical area of the brain, weight drop.	Mild. 2500 g weight, 70-degree angle dropped from 25 cm above the exposed skull, covered by a 1 mm thick metal plate. No motor or neurological deficits were observed. No major bleeding or necrosis.	30 dpi
Ren et al. 2024^77^	TBI decreases orexin and orexin receptors after injury.	Animals: Rats, Sprague-Dawley	6 weeks, 250–300 g	Males	Controlled cortical impact	Moderate: 4 m/sec, 150 msec dwell time, 2.8 mm impact depth.	14 dpi
Rowe et al. 2019^70^	No significant changes in orexin neuron number for any injured condition, including animals given a second injury.	Animals: Mice. C57BL/6 (Harlan laboratories)	Adolescent weight, 20–24 g	Males	Midline fluid percussion injury	Moderate: 1.4 atm.	7 dpi, 14 dpi and 28 dpi
Saber et al. 2020^71^	No significant changes in orexin neuron number after injury.	Animals: Mice, C57/BL6J (Jackson)	8 weeks old	Males and females	Midline fluid percussion injury	Moderate: Atm was matched to body weights to induce similar injury severity in sexes. Males 1.24 atm ± 0.01 and females 1.18 atm ± 0.004.	7 dpi
Skopin et al. 2015^73^	Significantly decreased orexin neurons in injured animals.	Animals: Rat, Sprague-Dawley	Adult, 310–330 g	Males	Lateral fluid percussion injury	Moderate: 1.9–2.2 atm.	29 dpi
Somach et al. 2023^83^	Increased size of afterhyperpolarization of action potentials in females after injury, reduced action potential threshold in males after injury. Decreased afferent excitatory signaling and increased afferent inhibitory signaling onto orexin neurons after injury.	Animals: Mice, C57/BL6J (Jackson)	6–8 weeks old	Males and females	Lateral fluid percussion injury	Mild: 1.4 to 1.6 atm.	6–10 dpi
Thomasy and Opp 2019^48^	TBI reduces the number of orexin neurons.	Animals: Mice. C57BL/6 and HCRT KO on C57BL/6 background	3–4 months old	Males	Controlled cortical impact	Moderate: 5.0 m/sec impact velocity, 100 msec dwell time, 1.0 mm impact depth.	32 dpi
Thomasy et al. 2017^45^	Mild TBI caused a significant decrease in orexin neurons 15 days after injury. Moderate TBI caused a significant decrease of orexin neurons 7 and 15 days after injury.	Animals: Mice, C57/BL6J (Jackson)	3–4 months old	Males	Controlled cortical impact	Mild: 0.5 mm impact depth. Moderate: 1.0 mm impact depth. For both: 5.0 m/sec impact velocity,100 msec dwell time.	7 dpi and 15 dpi
Vu et al. 2018^74^	No significant changes in orexin neuron number after injury for either injury method were used.	Animals: Mice, C57/BL6J (Jackson)	8 weeks old	Males	Mild CBI-closed head controlled cortical impact. mild blast-wave induced brain injury	Mild for both. Concussive brain injury parameters: 1.5 mm depth.	24 or 72 h post-injury
Willie et al. 2012^49^	Orexin neuropeptide in the hypothalamus and hippocampus decreased after injury. The number of orexin neurons increased after injury. Circadian fluctuation of orexin production was reduced in the hypothalamus after injury.	Animals: Mice, C57/BL6J (Jackson)	4–6 months old	Males	Controlled cortical impact	Moderately severe contusion: 5 m/sec, depth of 2.5 mm.	Microdialysis measurements were taken 5 days after impact, and immunohistochemistry was done 5 days after impact at the end of the experiment.
Zhong et al. 2015^76^	At 6 h post-injury, orexin receptors increased compared to sham-injured controls, then decreased at 12 h compared to sham-injured controls. Orexin increased at 6 h after injury compared to sham-injured controls.	Animals: Rat, Sprague-Dawley	Adult, 250–300 g	Males and females	Free fall weight-drop model	400 g weight dropped from a vertical height of 40–44 cm.	6 h, 12 h, 24 h

Severities of injury were defined by authors of studies and not written if not noted in their study.

Acronyms in table: TBI: traumatic brain injury; SD: standard deviation; CSF: cerebral spinal fluid; pg/mL: picograms per milliliter; g: grams; ms: milliseconds; mm: millimeters; Cm: centimeters; atm: atmospheres; psi: pounds per square inch; m/s or m/sec: meters per second; Dpi: days post injury; h, hr, or hrs: hours [post injury]; HCRT: hypocretin; KO: knockout.

Some studies measure orexin neuropeptide after injury rather than quantify orexin cell bodies. Several studies found an increase in orexin neuropeptide after TBI.^75,76^ In several other studies, including ours, there was a decrease in orexin after TBI.^49,77,78^ Many of these studies were done in adult Sprague-Dawley rats with the free-fall weight drop method of injury so they can be more readily compared. Though there may be other factors, one noticeable difference is the time of measurement post-injury. The studies that found an increase in orexin neuropeptide conducted their measurements between 6 and 24 h post-injury. The studies that found decreases in orexin neuropeptide conducted their measurements 24 h or longer after the injury. This could be an indication that there is an initial increase of orexin neuropeptide after an injury and then a decrease.

Two studies of orexin after TBI measured orexin at three acute time points which might give an indication if orexin neuropeptide first increases and then decreases. One study,^79^ found that orexin expression increased in general from 6 to 24 h after injury, though the highest orexin observed expression in their study was at 12 h after injury. It could be that orexin expression increases until 12 h and then decreases at 24. In one other study,^76^ measured orexin at 6, 12, and 24 h after injury. They observed significantly more orexin in injured animals compared to sham-injured animals at 6 h post-injury, but not at 12 or 24 h. The authors of Dong et al. 2018 discuss that the heightened orexin at 12 h may be because the measurement time is offset 12 h from the start of the study, which would change the circadian time that the orexin is measured. These two studies both do not control for circadian timing when they injure their animals. This may be because these studies were focused on measuring orexin after TBI as an outcome of nerve stimulation treatments. Orexin expression is dependent on circadian rhythm and timing is an important consideration when interpreting results from studies of orexin. In our study,^49^ we measured orexin over time and only saw decreased expression after injury, but all of our measurements were taken after 24 h post-injury. It would be useful to measure orexin in a time course in one model to know if the trend that is observed across multiple studies could be confirmed in one consistent study.

Some studies describe the functional outcomes of TBI on orexin neurons. In one of our previous studies, we reported that the animals with mild TBI had greater sleepiness in their waking period.^67^ We kept animals awake for several hours and then stained the orexin neurons with cFos, an immediate early gene that is a proxy for neural activity. Orexin neurons typically show an increase in cFos when animals are sleep deprived,^80,81^ but we found that injured animals had a smaller percentage of orexin neurons stained with cFos indicating less active orexin neurons after injury. In another study, we used electron microscopy to examine the glutamate and Gamma-Aminobutyric Acid (GABA) in presynaptic vesicles from neurons that synapse onto orexin neurons.^82^ We found that there was a decrease in the density of glutamate in the synapses on the dendrites of orexin neurons after TBI. We followed this result and found reduced glutamatergic activity in orexin neurons by measuring afferent input using whole-cell patch clamp electrophysiology.^83^ We also found that there was an increase in afferent GABAergic activity onto orexin neurons, which could account for a sum decrease in orexin neuron activity. These studies are the only functional studies of orexin neurons after TBI and suggest that TBI induces a reduction of orexin activity.

Orexin neurons project onto sites that express orexin receptors.^84,85^ There is evidence that the expression of orexin receptors is changed in response to disruptions such as inflammation, cardiac arrest, and ischemia,^86–88^ and it was studied if this phenomenon occurs after TBI. Several studies found an increase of orexin receptors compared to sham-injured animals after TBI,^75–79,89^ while other studies found a decrease of orexin receptors compared to sham-injured animals after TBI.^76,77,90,91^ These studies examined orexin receptor expression in different parts of the brain including TBI damaged cortex tissue,^77,78,89,90^ the hypothalamic region,^76,91^ or the prefrontal cortex.^75,79,91^ This could be one potential reason for the reported differences in receptor expression after injury. One study of orexin receptors,^76^ demonstrates a similar trend to the orexin neuropeptide expression, that there is an acute increase in orexin receptors followed by a decrease. Another study,^89^ also follows this trend and reports that orexin receptors peak at 24 h post-injury and decrease after, though this study only shows qualitative, not quantitative findings. There are some studies that do not match the trend of an acute increase of orexin neuropeptide and then a decrease. In one study,^91^ reports a decrease of orexin receptors soon after injury, and in one study,^78^ reports increased orexin receptor levels compared to sham-injured animals 3 days post-injury. In this particular study, there is both an increase in neuropeptide after injury and a decrease in orexin receptor levels, meaning that the two may not correspond with one another.

Mihara et al. 2011 went further and studied which cells had orexin receptor expression after injury. In their study, orexin receptor expression at 24 h post-injury occurred in microglia only. At 7 days after injury, orexin receptor expression was observed in microglia and neurons, but not in astrocytes. The group proposes that microglia might be part of an initial upregulation response of orexin receptors after injury. In an additional result related to the presence of orexin after injury, Mihara et al. 2011 observed that orexin neuronal fibers were more present in the area surrounding the injury at 24 h and 7 days after injury as compared to uninjured animals.^89^ Once again, this result is qualitative and not quantitative but this result suggests that orexin neuron fibers innervate a site of damage after TBI to potentially exert the effects of orexin in the area after injury. The changes in orexin receptor levels and fibers could cause short-term influences on the brain’s response to orexin neuropeptide following TBI.

## Women and Female Animals in TBI and Orexin Research

Finally, there is considerable evidence that sex is an important factor to consider in neuroscience research,^92–95^ and it is worth exploring this with respect to orexin, sleep, and TBI research. It has been observed that women accumulate sleep debt more rapidly than men do and might need more sleep to be functional.^96–98^ Interestingly, while women normally have longer and better sleep, they also report a higher incidence of sleep disorders.^99–102^ With regards to sleep after TBI, women often report having worse sleep outcomes after injury than men, and the female sex is considered to be a risk factor when measuring sleep disorders after injury.^103–107^ There are differences in orexin neuron populations between males and females. There is more of the precursor mRNA to orexin, prepro-orexin, in female animals,^108^ and higher levels of orexin in females.^109^ Previous research has shown that orexin neurons in males and females have differences in response to stress and disease.^110–112^ Despite this, the studies of TBI and orexin reviewed here have an underrepresentation of women and female animals. Out of the 25 studies reviewed here with outcomes related to orexin and TBI (Table 1), 9 include women or female animals. In the studies of human subjects, between 0 and 43% of the subjects were women. Out of the animal studies, 5 out of 19 use female animals. One study found no differences by sex with regard to orexin neurons themselves but did find differences in sleep parameters after injury.^71^ Another study found that male and female animals had differences in orexin neuron physiology after injury and that inhibitory activity might be more prominent in orexin neurons in females after injury.^83^ There is a growing awareness that it is important to include multiple sexes in research and this is especially the case where there is already evidence that sex may be relevant for sleep, TBI, and orexin research.

## Concluding Remarks

In humans and animal subjects, there is significant evidence that the orexin system is disrupted after TBI. This includes the gross level of the number of neurons, as well as the functional level of how individual orexin neurons behave in synaptic circuits. We have also discussed how orexin receptors are affected, a sign that after TBI, there may be a change in how the whole brain responds to orexin activity.

As with any group of TBI studies, the methods and models vary between each study which contributes to the range of outcomes observed. Differences in the ways that injuries are conducted, timing, and how results are gathered and interpreted undoubtedly contribute to differences in the data presented here. Each injury method has its features, advantages, and disadvantages which should all be considered when reviewing this data.

Most of the discussion presented in this article relates orexin to dysfunctions in sleep behavior after TBI. Arousal is not the only behavior related to orexin. Orexin is also associated with memory processing,^113–116^ motivation, reward,^117^ and addiction,^118^ which have been described as behaviors that are affected by TBI.^9,119–123^ Orexin is well known for its role in appetite stimulation. There are some reports that TBI can induce eating disruptions which can include both lack of interest in eating as well as hyperphagia.^119,124–126^ However, reports of orexin and TBI do not typically comment on the feeding aspects of orexin neurons and there are no animal model studies that incorporate orexin, TBI, arousal, and feeding together. This, along with other behaviors related to orexin that are not studied after TBI, could supplement studies of orexin that focus on sleep and arousal after injury.

## Transparency, Rigor, and Reproducibility

This review was not preregistered. Articles for this review were found using the National Institutes of Health PubMed database. Articles were found by searching for keywords such as TBI, head injury, TBI, orexin, orexin receptors, OXR, OX1R, and hypocretin. Articles were also found by searching citations of other articles related to orexin/hypocretin and TBI. The articles reviewed in this article that were found that directly related to findings of TBI and orexin/hypocretin are presented in Table 1 of this article. The review presented is a narrative review, the authors did not use a formalized search strategy such as that used in a systematic review and thus formalized search strategies are not presented within the article. The field of orexin/hypocretin and TBI is limited, and the authors felt a narrative review would cover the field sufficiently. One published article does have a finding related to orexin and TBI, Kousi et al. 2021, but the authors did not think there was sufficient control subject data presented in this work and did not include this study in Table 1. The authors do acknowledge that this study is within the scope of this review even if the results are not presented in the table of the lack of control subjects. The figure in this article was created in Inkscape.

## Abbreviations Used

atm: atmospheres
CSF: Cerebral Spinal Fluid
cm: centimeters
CCI: Controlled Cortical Impact
dpi: days post injury
EDS: Excessive Daytime Sleepiness
FPI: Fluid Percussion Injury
g: grams
GABA: Gamma-Aminobutyric Acid
h: hours
h, hr, or hrs: hours, post injury
HCRT: hypocretin
KO: Knockout
m/s or m/sec: meters per second
MCH: Melanin-Concentrating Hormone
mm: millimeters
ms: milliseconds
pg/mL: picograms per milliliter
psi: pounds per square inch
SD: standard deviation
TBIs: Traumatic Brain Injuries
